# Changes in Bile Acid Metabolism and Nutrient-Stimulated Hormone Responses After Malabsorptive Metabolic Surgery: RYGB Versus SADI-S

**DOI:** 10.3390/nu18203398

**Published:** 2026-10-16

**Authors:** Inés Jiménez-Varas, Isabel Ortega-Madueño, Angélica Larrad-Sainz, Ana Barabash-Bustelo, Andrés Sánchez-Pernaute, Pilar Matía-Martín, Natalia Pérez-Ferre, Ana María Ramos-Leví, Clara Marcuello Foncillas, Gemma Hernández-Núñez, María Victoria Sáez de Parayuelos, María Jesús Pérez-Castejón, Antonio Torres-García, Alfonso L. Calle-Pascual, Miguel A. Rubio-Herrera

**Affiliations:** 1 Department of Endocrinology and Nutrition, Hospital Clínico San Carlos, Instituto de Investigación Sanitaria del Hospital Clínico San Carlos (IdISSC), 28040 Madrid, Spain; i.jimenez.varas@gmail.com (I.J.-V.);; 2 Department of Clinical Analysis, Hospital Clínico San Carlos, Instituto de Investigación Sanitaria del Hospital Clínico San Carlos (IdISSC), 28040 Madrid, Spain; 3 Department of General Surgery, Hospital Clínico San Carlos, Instituto de Investigación Sanitaria del Hospital Clínico San Carlos (IdISSC), 28040 Madrid, Spain; 4 Faculty of Medicine, Universidad Complutense de Madrid, 28040 Madrid, Spain; 5 Centro de Investigación Biomédica en Red de Diabetes y Enfermedades Metabólicas Asociadas (CIBERDEM), Instituto de Salud Carlos III, 28029 Madrid, Spain; 6 Department of Nuclear Medicine, Hospital Clínico San Carlos, Instituto de Investigación Sanitaria del Hospital Clínico San Carlos (IdISSC), 28040 Madrid, Spain

**Keywords:** bile acids, bariatric-metabolic surgery, Roux-en-Y gastric bypass, SADI-S, gastrointestinal hormones, SeHCAT, nutritional status, mixed-meal test

## Abstract

**Background/Objectives:** Bariatric–metabolic surgery (BMS) improves obesity through neuroendocrine mechanisms involving bile acids (BAs) and nutrient-stimulated gastrointestinal hormones. However, the underlying pathophysiology involved remains poorly understood. BAs are essential for the digestion and absorption of dietary fat and fat-soluble vitamins and act as nutrient-responsive signaling molecules, so their handling after malabsorptive procedures is relevant both to metabolic outcomes and to nutritional status. **Methods:** We prospectively compared Roux-en-Y gastric bypass (RYGB) and single-anastomosis duodeno-ileal bypass with sleeve gastrectomy (SADI-S) in a single-center case-control study including 37 RYGB and 31 SADI-S patients with severe obesity and 19 non-obese controls. Before and one year after surgery, participants underwent a standardized mixed-meal test (nutrient challenge) with measurement of 14 individual BA fractions, insulin, GLP-1, ghrelin, FGF19, leptin, and adiponectin; bile acid malabsorption was assessed one year after surgery with the ^75^Se-SeHCAT test, and weight was followed for up to 5 years. **Results:** Fasting total BAs increased after both techniques, with no significant between-technique difference in total BA exposure (primary outcome). The techniques were associated with distinct BA profiles: fasting concentrations of conjugated BAs (GCA, GCDCA, and TDCA) were higher after SADI-S than after RYGB (FDR-adjusted q < 0.05), with a similar but more variable pattern in postprandial AUCs, whereas RYGB was characterized by a greater postprandial rise in the unconjugated secondary BA DCA. After surgery, FGF19 increased progressively during the mixed-meal test, peaking at 90 min, and its increase correlated with the rise in conjugated BAs, particularly in SADI-S. The postprandial insulin peak shifted earlier in both techniques, with a lower insulin AUC in SADI-S; GLP-1 responses were supraphysiological, and ghrelin increased post RYGB but was suppressed after SADI-S. Leptin decreased markedly, and in an exploratory analysis, its decrease was associated with weight-loss success at 3 years. Weight loss was greater with SADI-S at 1 year, but not significantly different at 3 and 5 years; in an exploratory analysis, the increase in conjugated BAs correlated negatively with recurrence weight gain from nadir at 5 years. The SeHCAT test showed no bile acid malabsorption in SADI-S and normal retention in 90.6% of RYGB patients. Despite routine supplementation, total protein, zinc, copper, and the vitamin A/RBP and vitamin E/cholesterol ratios decreased after both techniques. **Conclusions:** In conclusion, RYGB and SADI-S are associated with differentiated BA and hormonal profiles. The predominance of conjugated BAs after SADI-S, which we hypothesize may be related to its longer biliopancreatic limb, emerges as a potential candidate mechanism for weight maintenance that requires confirmation and was not accompanied by clinically relevant bile acid malabsorption; however, the decreases observed in several micronutrients support careful nutritional follow-up after both techniques. Clinical trial registration: ISRCTN81954082.

## 1. Introduction

Severe obesity is a chronic, multifactorial, and relapsing disease whose prevalence continues to rise worldwide. Obesity is associated with an increased risk of type 2 diabetes mellitus (T2DM), metabolic dysfunction-associated steatotic liver disease (MASLD), cardiovascular disease, and impaired quality of life [1].

Bariatric–metabolic surgery (BMS) has proven to be the most effective and durable treatment for severe obesity, achieving sustained body-weight reductions of more than 30% and long-term remission of comorbidities [2,3,4].

The BMS paradigm has evolved from a restrictive–malabsorptive approach toward a model involving a neuroendocrine component, in which both gastrointestinal hormones and bile acids (BAs), stimulated by nutrients in the distal intestine, play a central role in energy homeostasis [5]. The liver synthesizes primary BAs that, after conjugation with glycine or taurine, are converted into secondary BAs by the gut microbiota through deconjugation and 7α-dehydroxylation. BAs simultaneously stimulate the farnesoid X receptor (FXR) and the membrane receptor TGR5 (Takeda G protein-coupled receptor 5). The hepatic FXR ligand is fibroblast growth factor 19 (FGF19) [6], whereas TGR5 mediates GLP-1 secretion [7]. Together, BAs contribute to modulating glucose metabolism and energy expenditure [8,9].

In parallel, BMS modifies the profile of hormones involved in the regulation of weight and appetite control, such as leptin and adiponectin, secreted by adipose tissue. The adiponectin/leptin ratio has been proposed as an integrated biomarker of adipose tissue dysfunction and cardiometabolic risk [10,11], potentially reflecting overall metabolic improvement more accurately than either hormone alone. In addition, the relationship between the anatomical changes produced by different BMS techniques and the nutrient-stimulated gastrointestinal and pancreatic hormones (NuSH, Nutrient-Stimulated Hormones) is well established, thereby participating in the brain–intestine axis in the regulation of body weight.

The profound alteration of the enterohepatic circulation of BAs produced by BMS varies according to each technique [12]. BMS techniques differ markedly in their anatomy and degree of malabsorption. In this regard, proximal RYGB excludes the duodenum and proximal jejunum and entails a moderate degree of malabsorption, whereas the SADI-S technique (single-anastomosis duodeno-ileal bypass with sleeve gastrectomy) involves greater intestinal malabsorption by diverting food directly to the terminal ileum with a common channel, usually 250 cm from the ileocecal valve [13,14]. Despite this, the differences in the BA profile between the two techniques, both quantitative and qualitative, and their interaction with the gastrointestinal hormonal axis remain scarcely understood.

From a nutritional perspective, BAs are essential for the emulsification and absorption of dietary lipids and fat-soluble vitamins, and their secretion and reabsorption are tightly coupled to food intake. Malabsorptive procedures such as SADI-S, which shorten the intestinal segment exposed to nutrients and bile, have raised concerns about fat, protein, and micronutrient malabsorption and about bile acid loss, which could compromise nutritional status in the long term. Characterizing the BA response to a standardized nutrient challenge, together with BA retention and biochemical markers of nutritional status, may therefore help to understand how each technique modifies nutrient-stimulated signaling and to inform the nutritional follow-up of these patients.

In this context, the aim of this study was to compare the secretion of BAs and nutrient-stimulated gastrointestinal hormones after two BMS techniques with different degrees of intestinal malabsorption, RYGB and SADI-S, in response to a standardized mixed meal, together with bile acid malabsorption and biochemical markers of nutritional status one year after surgery.

## 2. Materials and Methods

### 2.1. Study Design and Population

This was a prospective, observational, analytical case-control study conducted at a single center. The study was approved by the Clinical Research Ethics Committee (code 16/229-E_BC, approved on 22 November 2016), conducted in accordance with the Declaration of Helsinki, and registered with the ISRCTN (number ISRCTN81954082). This is an observational analytical case-control study; the trial was registered retrospectively with the ISRCTN registry on 24 February 2018 after enrollment had begun in January 2017 under the approved protocol of the FIS-funded project PI16/01655.

Between January 2017 and January 2019, 74 patients with severe obesity (BMI ≥ 40 kg/m^2^ or ≥35 kg/m^2^ with major comorbidities) who were candidates for bariatric surgery were prospectively included, together with 19 healthy non-obese volunteers as a control group. Controls were recruited from among companions, relatives, and hospital staff and were required to be aged 18–65 years, with a BMI of 20–29.9 kg/m^2^ and no metabolic abnormalities (hypertension, glucose abnormalities, atherogenic dyslipidaemia, or altered liver profile). Exclusion criteria for controls were any therapeutic diet, inflammatory or immune disease, alcohol intake > 20 g/day, intake of probiotics, prebiotics, or bioactive fiber, recent antibiotic use, known liver, kidney or neurological disease, psychiatric illness or drug addiction, eating disorders, and pregnancy or breastfeeding. Because controls had no digestive disease or malabsorptive condition, bile acid malabsorption was not expected in this group. All participants provided written informed consent.

Surgical technique was allocated according to the center’s clinical protocol: RYGB for patients with a BMI < 45–50 kg/m^2^ or with gastroesophageal reflux and/or hiatal hernia, and SADI-S for patients with a BMI > 45 kg/m^2^ without gastroesophageal disease. All procedures were performed laparoscopically, without serious perioperative complications or mortality. RYGB was standardized with a 75 cm biliopancreatic limb and a 150 cm alimentary limb. SADI-S was performed with a 250 cm common channel from the ileocecal valve. In RYGB, the gastric pouch was approximately 20–30 mL, although its exact size could vary depending on the surgeon; in SADI-S, the vertical sleeve gastrectomy was calibrated over a 54 Fr bougie.

### 2.2. Dynamic Mixed-Meal Test

Laboratory evaluations were performed at two time points: before surgery (T0) and one year after surgery (T1). The control group was assessed at a single time point. In all cases, the protocol used was standardized as follows. The mixed-meal test (MMT) was performed after a 12 h overnight fast. The patient was placed comfortably on a bed in the supine position, and an antecubital vein was cannulated for blood sample collection. The patient was instructed to take a 200mL commercial liquid preparation (T-DIET 10/2^®^, Vegenat S.A., Badajoz, Spain) containing 400 kcal, with a macronutrient composition of 45% carbohydrates, 20% protein, and 35% fat, in less than 10 min. Venous blood samples were drawn at minutes 0, 30, 60, 90, and 120 in serum tubes with clot activator (REF 367896, BD Vacutainer^®^, Plymouth, UK) and in plasma tubes containing 15% K3E and 250 KIU aprotinin (REF 361017, BD Vacutainer^®^, Plymouth, UK). Then, samples were centrifuged at 1700× *g* for 10 min at 4 °C, within a maximum of 1 h after collection. The serum and plasma obtained were separated into aliquots and frozen at –80 °C until subsequent analysis.

### 2.3. Laboratory Evaluations

All laboratory evaluations were performed at the two study time points for patients who underwent surgery (before surgery and one year after surgery), and at a single time point for the non-obese controls. Prior to the mixed-meal test, all participants underwent a fasting blood analysis including complete blood count, fasting glucose, glycated hemoglobin (HbA1c), lipid panel (total cholesterol, HDL-C, LDL-C, triglycerides), hepatic panel (ALT, AST, GGT, alkaline phosphatase), renal panel, serum proteins, ferritin, vitamins (A, E, D, and B12), folate, parathyroid hormone (PTH), zinc, copper, insulin, and homeostatic model assessment of insulin resistance (HOMA-IR). All determinations were processed in the Central Laboratory of the Hospital using the following methodologies. General biochemistry (lipid, hepatic, renal, and protein panels) was analyzed on a Beckman Coulter AU5800 autoanalyzer (Beckman Coulter, Brea, CA, USA). HbA1c was determined by high-performance liquid chromatography (HPLC) on a Tosoh G8 analyzer (Tosoh Bioscience, Tokyo, Japan). Insulin was quantified by chemiluminescence immunoassay (CLIA) on a DxI 9000 Access analyzer (Beckman Coulter) with an analytical sensitivity of 0.03 µIU/mL. Ferritin was determined on an Atellica CI autoanalyzer (Siemens Healthineers, Erlangen, Germany). Vitamin B12 and folate were measured by CLIA on the DxI 9000 Access analyzer (Beckman Coulter); 25-hydroxyvitamin D was quantified by CLIA on an Alinity ci analyzer (Abbott Laboratories, Abbott Park, IL, USA). Intact PTH was determined by electrochemiluminescence immunoassay (ECLIA) on a Cobas e411 analyzer (Roche Diagnostics, Mannheim, Germany). Vitamins A and E were quantified by HPLC. Zinc and copper were determined by atomic absorption spectrometry. HOMA-IR was calculated as: fasting insulin (µIU/mL) × fasting glucose (mg/dL)/405.

### 2.4. Determination of Gastrointestinal, Pancreatic, and Adipose Tissue Hormones

The following parameters were measured at each of the 5 time points of the dynamic curve: total GLP-1 (Millipore^®^ ELISA, Darmstadt, Germany), total ghrelin (DRG^®^ ELISA, Springfield, NJ, USA), insulin (measured in serum by chemiluminescence immunoassay [CLIA] on a DxI 9000 Access analyzer; Beckman Coulter, Brea, CA, USA; analytical sensitivity 0.03 µIU/mL), and FGF19 (DRG^®^ sandwich ELISA), whereas leptin (DRG^®^ ELISA) and adiponectin (Biovendor^®^ ELISA, Brno, Czech Republic) were measured at a single baseline point (time 0).

### 2.5. Determination of Bile Acids

Fourteen individual bile acid (BA) fractions were quantified by ultra-high-performance liquid chromatography coupled with mass spectrometry (UHPLC-MS, LCMS-2020 system, Shimadzu^®^, Kyoto, Japan) after deproteinization with acetonitrile. The full names, abbreviations, and classifications of the 14 BAs analyzed are detailed in Table 1. Fasting concentrations, peak secretion, and the area under the curve (AUC) were calculated.

The area under curve (AUC) for each parameter measured during the mixed-meal test at the different time points (0, 30, 60, 90, and 120 min) was estimated using the trapezoidal method.

### 2.6. ^75^Se-SeHCAT Isotopic Test

To determine the potential degree of bile acid malabsorption one year after surgery, we performed an isotopic study using oral administration of the radiopharmaceutical tauroselcholic acid (75Se) (SeHCAT, GE Healthcare Bio-Sciences, SAU, Madrid, Spain) [15]. Tauroselcholic acid is a bile acid analog with a physiological behavior identical to that of conjugated BAs. After oral ingestion, in a fasting state, of a 0.37 MBq (0.01 mCi) capsule, abdominal activity was measured with a dual-head gamma camera over the abdominal region for 5 min. Seven days later, the percentage of radiopharmaceutical retention in the abdomen was measured using the same procedure. Retention at day 7 was classified into four mutually exclusive categories: normal (>15%), mild (>10% to 15%), moderate (5% to 10%), and severe (<5%) bile acid malabsorption [16,17].

Controls did not undergo this test, since bile acid malabsorption was not expected in the absence of digestive disease or intestinal surgery.

### 2.7. Weight Follow-Up Study

Weight, height, and BMI data were recorded at baseline and after 1, 3, and 5 years of follow-up after BMS. The percentage of weight loss (%WL = [(initial weight − current weight)/initial weight] × 100) and the percentage of recurrence weight gain from nadir (RWG = [(final weight − nadir weight)/(nadir weight − initial weight)] × 100) were calculated.

### 2.8. Statistical Analysis

The sample size of the FIS-funded protocol (PI16/01655) was calculated to assess changes in total BA concentrations according to surgical technique based on the standard deviations of total BAs after surgery reported by Jahansouz et al. [18]: 32 subjects per surgical group were required to detect a difference of ≥1.92 μmol/L (α = 0.05; β = 0.20; two-sided; SD = 3.62; estimated dropout rate 10%). Accordingly, the change in total BAs according to surgical technique was considered the primary outcome of the present analysis. No separate power calculation was performed for individual BA fractions or hormones; all other analyses (individual BA fractions, gastrointestinal, pancreatic and adipose-tissue hormones, ^75^Se-SeHCAT, weight and correlation analyses) are considered secondary or exploratory. Continuous variables are expressed as mean ± standard deviation (SD) or median with interquartile range (IQR) according to whether they were normally or non-normally distributed; normality was assessed with the Kolmogorov–Smirnov test. Within-group comparisons before vs. one year after surgery were performed with paired tests (Wilcoxon signed-rank test or paired Student’s *t*-test, as appropriate). Between-group comparisons (RYGB vs. SADI-S, and each surgical group vs. controls) were performed with the Mann–Whitney U test or the unpaired Student’s *t*-test, as appropriate, and categorical variables were compared with the χ^2^ test or Fisher’s exact test. Correlations were evaluated using Pearson and Spearman coefficients. To account for multiple comparisons across the 14 individual BA fractions, the Benjamini–Hochberg false discovery rate (FDR) procedure was applied within each family of 14 tests (within-group pre–post comparisons in RYGB and in SADI-S, and between-technique comparisons at one year), separately for fasting concentrations and postprandial AUCs. FDR-adjusted q values are reported alongside the corresponding p values in the Results tables, and q < 0.05 was considered robust to multiple testing. For the remaining analyses, nominal *p* values are reported, and these analyses should be interpreted as exploratory. An exploratory binary logistic regression analysis was performed to assess potential predictors of weight-loss success at 3 and 5 years, defined as a weight loss ≥ 30% of initial weight [4]. The percentage changes at one year in the AUC of gastrointestinal and pancreatic hormones and of BA fractions, as well as in leptin and adiponectin, were explored as candidate predictors. Given the moderate sample size, this analysis was considered hypothesis-generating. All analyses were performed on a complete-case basis at each time point, without imputation of missing data. Statistical significance was set at *p* < 0.05 (two-sided). Analyses were performed with SPSS software version 26.0 (IBM Corp., Armonk, NY, USA); FDR-adjusted q values were calculated with the statsmodels package (version 0.15.0) in Python (version 3.11).

## 3. Results

One year after surgery, the evolution of biochemical parameters is summarized in Table 2. Fasting glucose, HbA1c, and HOMA-IR decreased significantly in both techniques, reaching values close to those of the control group. The lipid profile improved significantly after both procedures, with reductions in total cholesterol, LDL-C, and triglycerides, and an increase in HDL-C. Regarding the hepatic profile, no significant changes were observed in AST, ALT, or alkaline phosphatase, whereas GGT decreased significantly after both techniques. Total protein decreased significantly after both techniques and albumin after RYGB, remaining within the normal reference range. Iron levels remained stable after RYGB and increased significantly after SADI-S, without differences in ferritin. Folate increased significantly after RYGB but not after SADI-S. Vitamin B12 concentrations were preserved in both groups; 25-hydroxyvitamin D increased significantly after RYGB, whereas no significant change was observed in SADI-S. The vitamin A/RBP ratio and the vitamin E/total cholesterol ratio decreased significantly after both techniques, as did zinc and copper concentrations. PTH did not change significantly in either group. All patients received oral micronutrient supplementation according to the standard postoperative protocol.

### 3.1. Patients’ Baseline Characteristics

Of the 74 initial patients, 40 underwent RYGB and 34 were eligible for SADI-S. Six patients (8.1%), three in each group, discontinued follow-up before the one-year assessment, leaving 37 RYGB patients and 31 SADI-S patients finally evaluable. The planned sample size (32 patients per group) was therefore reached in the RYGB group and nearly reached in the SADI-S group (31 evaluable patients).

Patients in the SADI-S group had a significantly higher BMI than those in the RYGB group (48.0 ± 4.72 vs. 44.1 ± 4.28 kg/m^2^; *p* < 0.001), with no significant difference in age (45.4 ± 9.85 vs. 50.0 ± 9.25 years; *p* = 0.051). Women represented 84% of participants in both groups (Table 3).

The proportion of patients with T2DM was numerically higher in the RYGB group, without statistically significant differences between techniques. The remaining comorbidities and demographic characteristics were comparable between the two techniques.

In the control group, mean age was 48.4 ± 9.53 years, 95% were women, and the mean BMI was 23 ± 2.43 kg/m^2^.

#### Weight Follow-Up

One year after BMS, the percentage of weight loss (%WL) was significantly higher in the SADI-S group than in RYGB (41.3 ± 12.8% vs. 35.4 ± 7.77%; *p* = 0.028). The differences in %WL between techniques at 3 and 5 years were not statistically significant, and both techniques maintained a mean weight loss above 30% at 5 years of follow-up (Table 4). Follow-up rates were 100%, 97%, and 85% at 1, 3, and 5 years, respectively (at 5 years, 86% in RYGB and 84% in SADI-S), and analyses at each time point were performed on a complete-case basis. The final BMI was comparable between techniques at 1, 3, and 5 years, although a numerically greater proportion of SADI-S patients maintained weight-loss percentages ≥ 30% and ≥35%.

The percentage of recurrence weight gain (RWG) from nadir at 5 years was 14.9% (95% CI: 3.6–18.1) for RYGB and 10.4% (95% CI: −0.6–19.1) for SADI-S, with no statistically significant difference between the two techniques.

### 3.2. Changes in Gastrointestinal, Pancreatic, and Peripheral Hormones

#### 3.2.1. Insulin Concentrations

**Figure 1:**
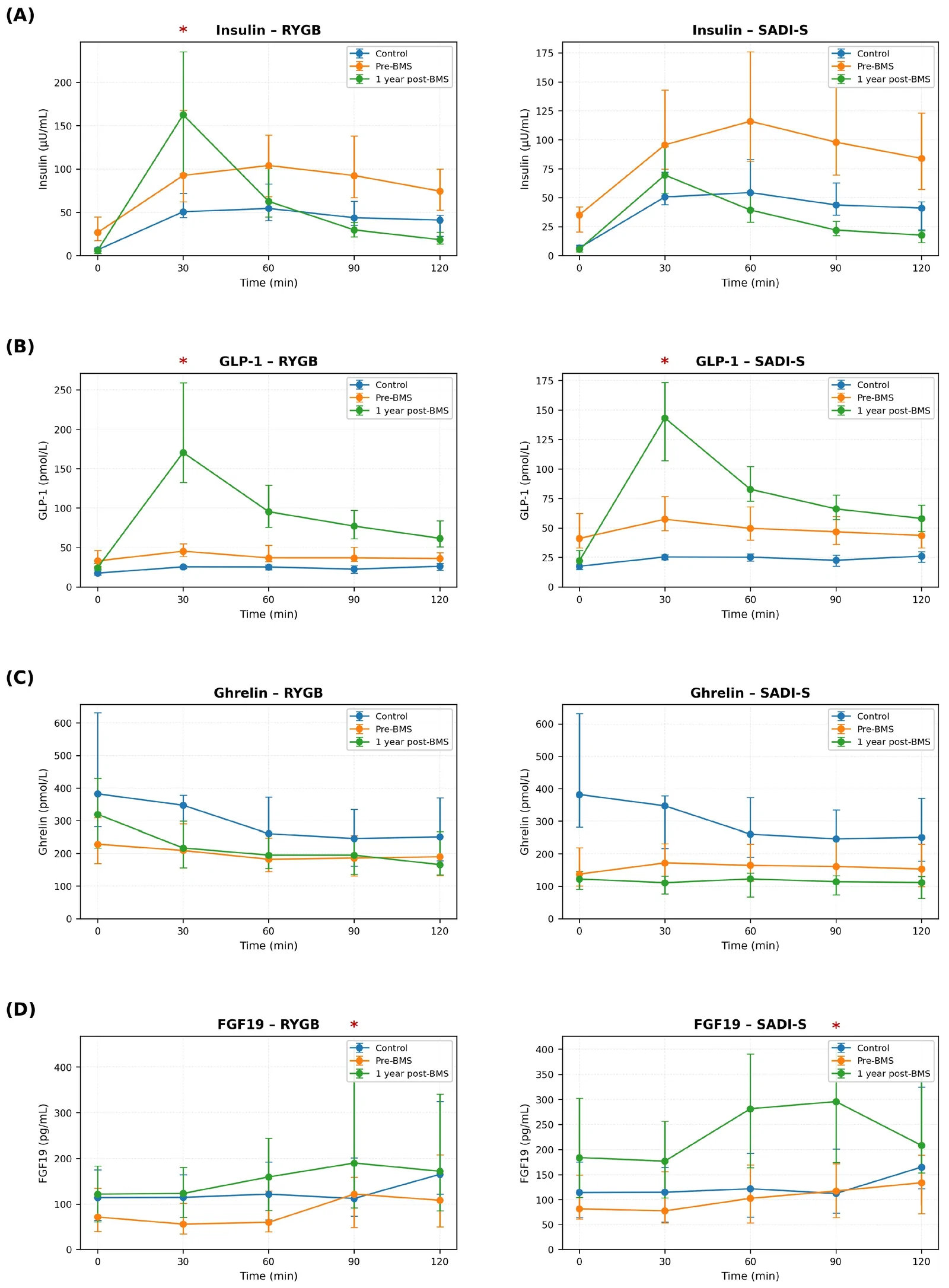
Postprandial hormone responses to the mixed-meal test before and after RYGB and SADI-S, compared with the control group. (**A**) Insulin, (**B**) GLP-1, (**C**) Ghrelin, (**D**) FGF19. * Significant differences (*p* < 0.01): insulin at 30 min in the RYGB group; GLP-1 at 30 min in both groups; and FGF19 compared with the control group.

Fasting insulin concentrations were significantly higher in patients with obesity than in the control group (Table 5) and normalized after both surgical techniques, without significant differences between the procedures (RYGB: 7.6 ± 7.0; SADI-S: 5.81 ± 3.45 μIU/mL; *p* < 0.01 for the decrease vs. before surgery in both techniques). After the mixed-meal test, the insulin peak shifted from minute 60 (before surgery) to minute 30 (after surgery), with supraphysiological elevations of 21-fold the baseline value in RYGB and 14-fold in SADI-S, reaching statistical significance only in RYGB (*p* < 0.01) (Figure 1A). The insulin AUC decreased after both surgeries, reaching statistical significance in SADI-S (5092 vs. 12,546 μIU·mL^−1^·min before surgery; *p* < 0.01).

#### 3.2.2. GLP-1

GLP-1 responses were similarly supraphysiological in both techniques (6-fold baseline in RYGB; 5.5-fold in SADI-S) (Figure 1B), with a significantly higher AUC in RYGB (12,542 vs. 10,540 pmol·L^−1^·min; *p* < 0.05). The GLP-1 peak at 30 min one year after BMS showed a weak positive correlation with %WL at 3 years in SADI-S (r = 0.36; *p* = 0.049); given its marginal significance and the number of correlations tested, this finding should be considered exploratory.

#### 3.2.3. Ghrelin

Ghrelin showed opposite behaviors between techniques: RYGB produced a significant increase in fasting concentrations (from 259.4 ± 141.2 to 349 ± 205.6 pmol/L; *p* < 0.05), whereas SADI-S showed a decrease that did not reach statistical significance, with a significantly lower AUC (14,261 vs. 25,531 pmol·L^−1^·min before surgery; *p* < 0.01) (Figure 1C). Post-surgical concentrations in SADI-S were significantly lower than in RYGB (*p* < 0.01).

#### 3.2.4. Fibroblast Growth Factor 19 (FGF19)

After surgery, the FGF19 response to the MMT was inverted compared with controls: whereas controls showed an initial decrease at 30 min, FGF19 increased progressively after both techniques, with an early rise at 30 min in RYGB and a peak at 90 min in both groups (Figure 1D). The FGF19 AUC increased significantly compared to before surgery in both techniques (RYGB: from 16,835 to 29,970 ± 38,400, *p* < 0.05; SADI-S: to 34,723 ± 22,564 pg·mL^−1^·min, *p* < 0.01), reaching values higher than the control group only in SADI-S (*p* < 0.05), with no differences between techniques.

#### 3.2.5. Leptin and Adiponectin

In the exploratory logistic regression analysis (outcome: weight loss ≥ 30% of initial weight), the percentage decrease in leptin at one year was associated with weight-loss success at 3 years (RYGB: OR = 0.91, 95% CI 0.84–0.98, *p* = 0.015; SADI-S: OR = 0.95, 95% CI 0.91–0.99, *p* = 0.045), but not at 5 years. Given the sample size, this association should be considered hypothesis-generating and requires confirmation in larger cohorts.

### 3.3. Changes in Bile Acids

#### 3.3.1. Total, Primary, and Secondary Bile Acids

**Figure 2:**
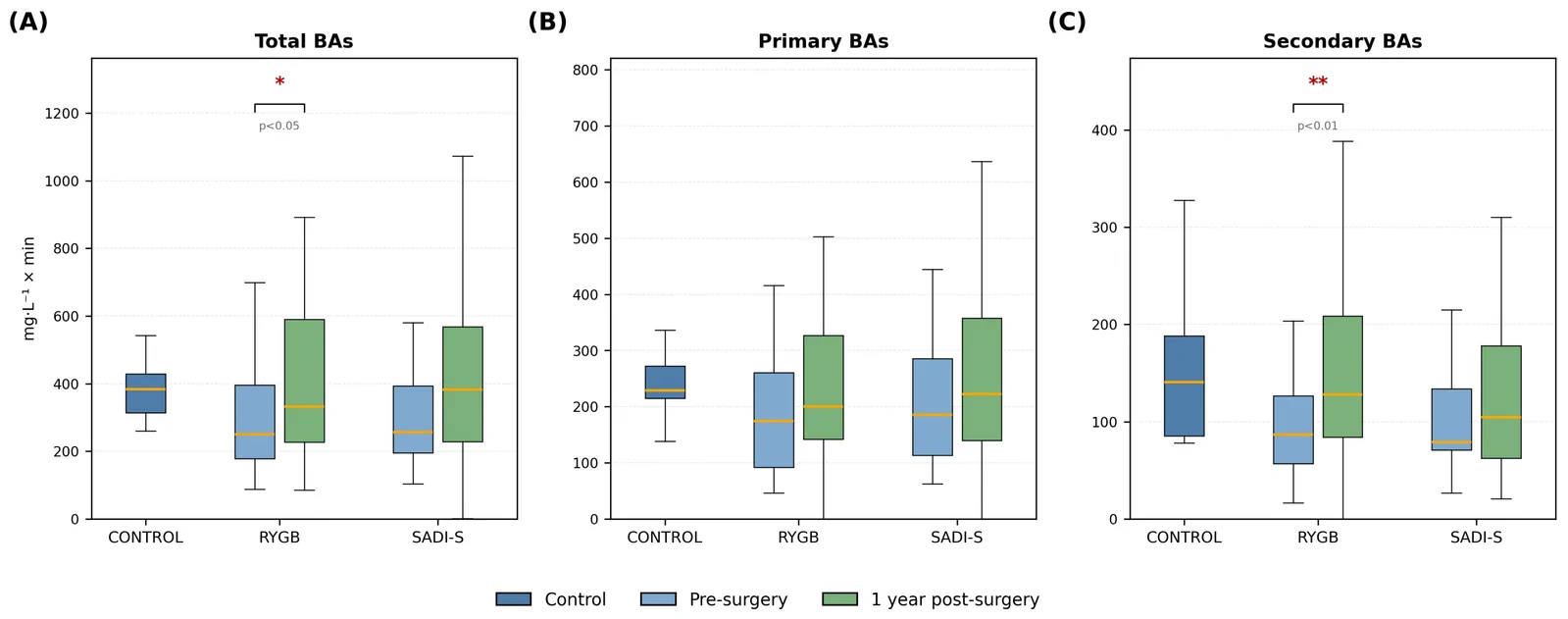
Postprandial response (AUC) of total, primary, and secondary bile acids in the control group and in individuals with obesity before and one year after bariatric–metabolic surgery. (**A**) Total BAs, (**B**) Primary BAs, (**C**) Secondary BAs. Boxes represent median and interquartile range; whiskers extend to non-outlier extremes. Significance is indicated by asterisks: * *p* < 0.05, ** *p* < 0.01. Asterisks indicate nominal *p* values. RYGB, Roux-en-Y gastric bypass; SADI-S, single-anastomosis duodeno-ileal bypass with sleeve gastrectomy; AUC, area under the curve.

Fasting concentrations of total, primary, and secondary BAs all increased significantly one year after surgery in both techniques (all *p* ≤ 0.004), reaching values comparable to those of the control group. Before BMS, the postprandial AUC of total and secondary BAs was significantly lower in individuals with obesity than in the control group, with no differences in primary BAs (Table 6). One year after surgery, both techniques increased the postprandial AUC of total BAs toward values close to those of the control group, reaching statistical significance in RYGB but not in SADI-S. This increase was more pronounced in the secondary BA fraction, again reaching significance only in the RYGB group. Regarding the primary outcome (change in total BAs according to surgical technique), no significant differences were observed between techniques in the AUC of total BAs, nor in those of primary or secondary BAs (Table 6, Figure 2). However, when individual fractions were analyzed, differences between techniques emerged mainly in the fasting concentrations of conjugated BAs, which were higher after SADI-S (Table 7), suggesting a qualitative rather than a quantitative shift in the BA profile. After FDR correction (Table 7 and Table 8), the between-technique differences that remained significant were the higher fasting concentrations of GCA, GCDCA, and TDCA in SADI-S; for postprandial AUCs, no between-technique difference remained significant after correction, the largest being the higher DCA AUC in RYGB (q = 0.054). Within-group changes that remained significant after FDR correction included the increases in fasting GCA, GCDCA, and GDCA after both techniques and the postprandial increase in DCA after RYGB (q < 0.001).

#### 3.3.2. Unconjugated Primary Bile Acids: CA and CDCA

**Figure 3:**
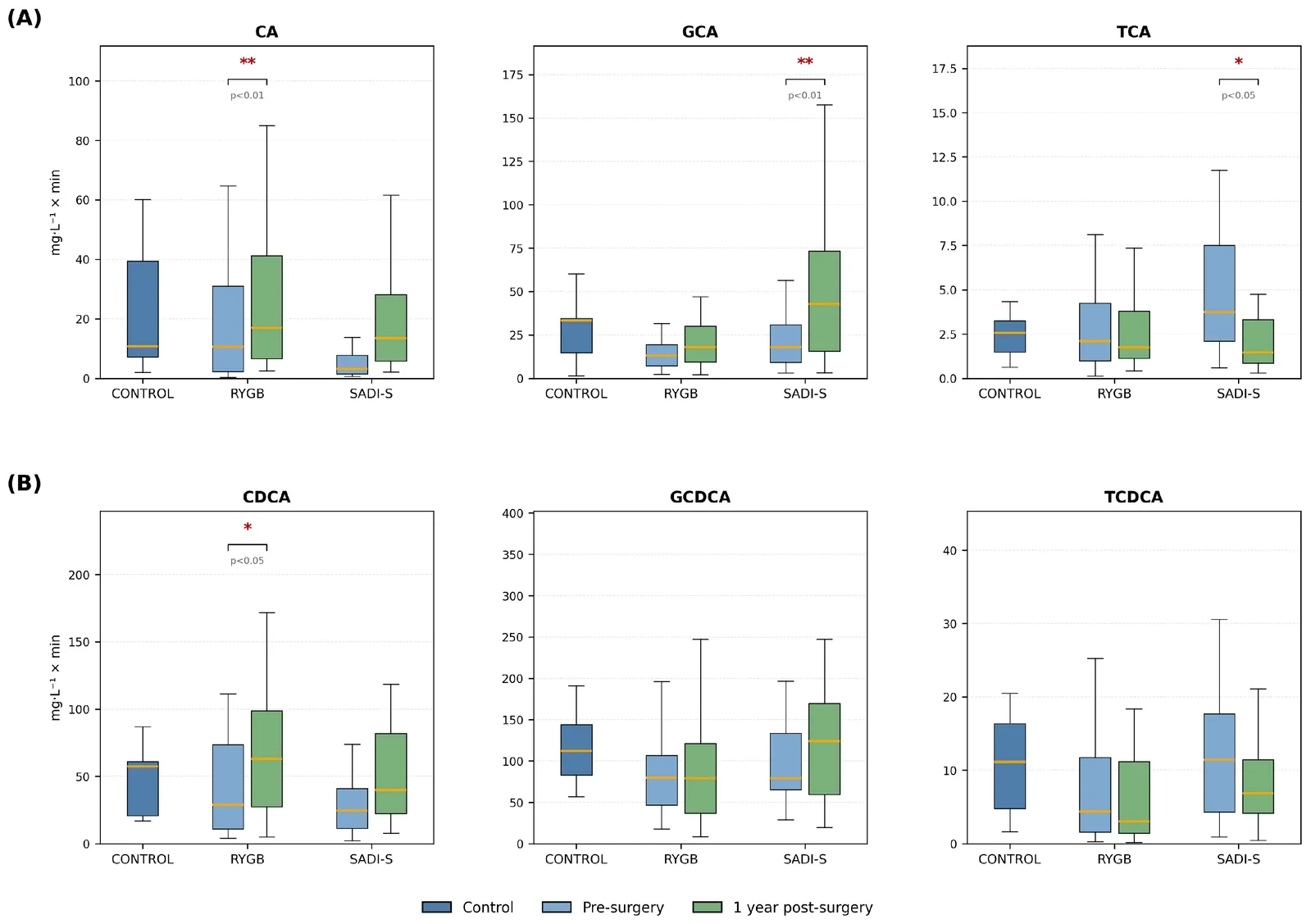
Postprandial response (AUC) of individual primary bile acids in the control group and in individuals with obesity before and one year after bariatric–metabolic surgery. (**A**) Cholic acid (CA), glycocholic acid (GCA), and taurocholic acid (TCA). (**B**) Chenodeoxycholic acid (CDCA), glycochenodeoxycholic acid (GCDCA), and taurochenodeoxycholic acid (TCDCA). Boxes represent median and interquartile range; whiskers extend to non-outlier extremes. Significance is indicated by asterisks: * *p* < 0.05, ** *p* < 0.01. Asterisks indicate nominal *p* values; FDR-adjusted q values for individual BA fractions are reported in Table 7 and Table 8. RYGB, Roux-en-Y gastric bypass; SADI-S, single-anastomosis duodeno-ileal bypass with sleeve gastrectomy; AUC, area under the curve.

The behavior of cholic acid (CA) and chenodeoxycholic acid (CDCA) was similar between the two techniques. Both fractions, reduced in patients with obesity compared to the control group, increased after BMS, significantly in RYGB (fasting CA and CDCA and the CA AUC remained significant after FDR correction) and as a non-significant trend in SADI-S (Table 8, Figure 3).

#### 3.3.3. Conjugated Primary Bile Acids: GCA, GCDCA, TCDCA, and TCA

The conjugated primary fractions showed a technique-differentiated pattern, with higher post-surgical values in SADI-S, mainly in fasting concentrations. After BMS, fasting concentrations of GCA and GCDCA increased significantly after both techniques, and TCDCA only after SADI-S; fasting GCA, GCDCA, and TCDCA were higher in SADI-S than in RYGB, and the differences in GCA and GCDCA remained significant after FDR correction. For the AUCs, GCA increased significantly only in SADI-S, the GCA and TCDCA AUCs were higher in SADI-S than in RYGB, and the TCA AUC decreased significantly in SADI-S; none of these postprandial differences remained significant after FDR correction (Table 7, Figure 3).

#### 3.3.4. Unconjugated Secondary Bile Acids: DCA, LCA, and UDCA

**Figure 4:**
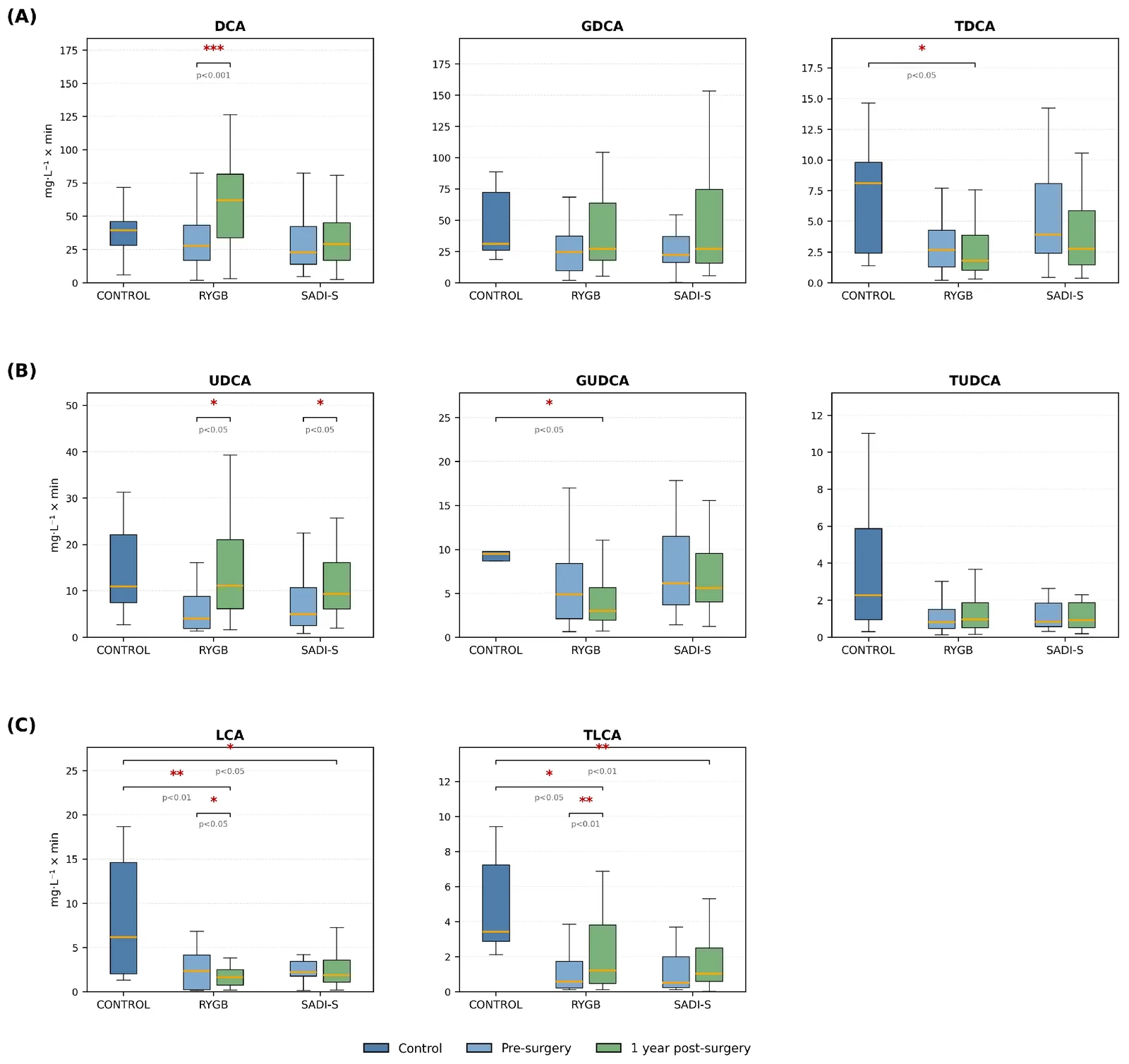
Postprandial response (AUC) of individual secondary bile acids in the control group and in individuals with obesity before and one year after bariatric–metabolic surgery. (**A**) Deoxycholic acid (DCA), glycodeoxycholic acid (GDCA), and taurodeoxycholic acid (TDCA). (**B**) Ursodeoxycholic acid (UDCA), glycoursodeoxycholic acid (GUDCA), and tauroursodeoxycholic acid (TUDCA). (**C**) Lithocholic acid (LCA) and taurolithocholic acid (TLCA). Boxes represent median and interquartile range; whiskers extend to non-outlier extremes. Significance is indicated by asterisks: * *p* < 0.05, ** *p* < 0.01, *** *p* < 0.001. Asterisks indicate nominal *p* values; FDR-adjusted q values for individual BA fractions are reported in Table 7 and Table 8. RYGB, Roux-en-Y gastric bypass; SADI-S, single-anastomosis duodeno-ileal bypass with sleeve gastrectomy; AUC, area under the curve.

The postprandial AUC of deoxycholic acid (DCA) increased significantly only in patients undergoing RYGB (q < 0.001 after FDR correction), reaching values numerically higher than the control group and higher than the SADI-S group (*p* = 0.004; q = 0.054), which constitutes one of the most marked differences between techniques. The UDCA AUC increased significantly after both techniques to values comparable to the control, whereas that of LCA (the most cytotoxic BA) decreased significantly in RYGB, with values below the control group after both techniques (Table 8, Figure 4).

#### 3.3.5. Conjugated Secondary Bile Acids: GUDCA, GDCA, TDCA, TLCA, and TUDCA

As with the conjugated primary fractions, fasting concentrations of GDCA and GUDCA increased significantly after both techniques, and TDCA only after SADI-S; fasting GUDCA and TDCA were higher in SADI-S than in RYGB, and the difference in TDCA remained significant after FDR correction. These differences between techniques were observed mainly in the fasting state (time 0 of the MMT) since the postprandial AUCs showed hardly any significant changes (the GUDCA AUC was higher in SADI-S than in RYGB, *p* = 0.014, but not after FDR correction). Fasting TLCA increased only in RYGB, and its AUC increased significantly in RYGB, remaining below the control group in both techniques (Table 7, Figure 4).

#### 3.3.6. Correlations Between Bile Acid Concentrations and Weight Outcomes

The analysis between the percentage changes in the AUC of the different BA fractions and the percentage of weight loss (%WL) at 1, 3, and 5 years showed no statistically significant associations at any time point for any BA fraction, either in the overall BMS group or in the subgroups by surgical technique.

However, in an exploratory analysis of recurrence weight gain from nadir (RWG) as a continuous variable, a negative correlation was observed between the percentage change in the AUC of conjugated BAs and RWG in the overall BMS group (Spearman: rho = −0.37; *p* = 0.016; *n* = 41). This finding was particularly evident in the SADI-S subgroup (rho = −0.45; *p* = 0.03; *n* = 22), whereas it did not reach statistical significance in RYGB (*n* = 19). Given the number of correlations tested, the moderate sample size and the absence of multiplicity correction, these associations should be considered exploratory.

In addition, a strong-to-moderate positive correlation was observed between the percentage change in the FGF19 AUC and the changes in the AUC of total, primary, and conjugated BAs, both in the overall group (r = 0.55; *p* < 0.01 for total BAs; r = 0.56; *p* < 0.01 for conjugated BAs) and specifically in the SADI-S subgroup (r = 0.73; *p* < 0.01 for total BAs; r = 0.77; *p* < 0.01 for conjugated BAs). This association was not statistically significant in the RYGB subgroup.

### 3.4. Assessment of Bile Acid Malabsorption Using the ^75^Se-SeHCAT Test

The ^75^Se-SeHCAT test was performed in 32 patients from the RYGB group and 29 from the SADI-S group one year after surgery (Table 9). The results showed that 90.6% of RYGB patients and 100% of SADI-S patients had normal retention (>15%). In the RYGB group, 2 patients (6.25%) had mild (>10% to 15%) and 1 patient (3.1%) had moderate (5% to 10%) bile acid malabsorption, and no patient had severe malabsorption (<5%); none of these patients reported clinically relevant malabsorption symptoms. No patient in the SADI-S group had bile acid malabsorption of any degree.

## 4. Discussion

BMS has proved to be the most effective therapeutic strategy for the long-term treatment of severe obesity, and its efficacy extends beyond the restrictive–malabsorptive effect, involving profound neuroendocrine changes mediated by nutrient-stimulated gastrointestinal hormones and the concomitant secretion of BAs. To our knowledge, this is the first study to characterize the postprandial profile of individual BA fractions in humans after SADI-S, with paired pre- and post-surgical assessment, alongside the gastrointestinal, pancreatic, and adipose-tissue hormone response and an objective evaluation of bile acid malabsorption by ^75^Se-SeHCAT. Previous human data after this technique are limited to total BA concentrations in a small cross-sectional series [19], whereas individual BA profiling has been reported only after classic duodenal switch under fasting conditions [20] or in animal models. This design allowed us to move beyond the description of total BA concentrations that has dominated the field and to describe technique-associated BA signatures that may help generate hypotheses on how each procedure achieves and maintains weight loss.

The central hypothesis of this work was to determine whether different BMS techniques, with different degrees of intestinal malabsorption, could modify BA metabolism differentially, thereby helping to explain the outcomes regarding weight loss and its long-term maintenance.

The most novel finding of our study is the observation of a BA signature associated with each technique. After RYGB, we observed a significant increase in total BAs, especially in unconjugated secondary BAs such as DCA. After SADI-S, in contrast, the profile was characterized by higher fasting concentrations of conjugated BAs. We hypothesize that the longer biliopancreatic limb of SADI-S could produce a distinct enterohepatic dynamic. Anatomically, the confluence of the biliopancreatic and alimentary limbs occurs closer to the terminal ileum than in RYGB, effectively shortening the enterohepatic circuit and enabling earlier BA reabsorption within the biliopancreatic limb itself. This accelerated recycling would promote a compensatory increase in hepatic conjugation [21,22], which would be consistent with the higher fasting concentrations of conjugated BAs observed after SADI-S. It should be noted that these differences were observed mainly in fasting concentrations, whereas postprandial AUCs showed the same direction but greater variability and fewer significant differences; therefore, our data support a technique-associated difference in fasting conjugated BA levels rather than a distinct postprandial signature. After FDR correction, the higher fasting GCA, GCDCA, and TDCA concentrations in SADI-S and the postprandial DCA increase in RYGB remained significant, whereas differences that did not remain significant after correction should be interpreted with caution. Our SeHCAT results, which showed normal retention of this conjugated BA analog in all SADI-S patients, are compatible with this interpretation since they argue against bile acid malabsorption as an alternative explanation. Importantly, this model is a working hypothesis based on the observed associations and on prior literature, and it was not directly tested in the present study: we did not measure intestinal transit, ileal BA reabsorption rates, hepatic conjugation activity, de novo BA synthesis (e.g., C4), or FXR/TGR5 receptor activation, which would be required to confirm it. Moreover, given the non-randomized design and the baseline differences between groups (higher BMI in SADI-S and numerically higher prevalence of T2DM in RYGB), the observed differences cannot be causally attributed to limb length or to any other specific anatomical feature. Published data on BA behavior specifically after SADI-S are scarce and heterogeneous, differing in sample size, follow-up, and analytical methodology [19,20], and this predominance of conjugated forms has not, to our knowledge, been previously described for this technique. Conjugated BAs are more potent ligands of the TGR5 receptor than their unconjugated forms, which could contribute to a more sustained enteroendocrine stimulation and inhibition of hepatic lipogenesis [7]. Although the early postprandial GLP-1 peak was more pronounced after RYGB—likely reflecting the rapid gastric emptying secondary to the absence of a pylorus—the higher availability of conjugated BAs in SADI-S may provide a longer-term amplification of TGR5-mediated signaling, which could plausibly contribute to the greater weight loss observed at one year with this technique. Notably, this pattern mirrors that recently reported in a randomized trial comparing RYGB with one-anastomosis gastric bypass, in which the technique with the longer biliopancreatic limb also increased conjugated primary BAs, whereas RYGB increased unconjugated secondary BAs, mainly DCA [23]. The convergence of both studies is consistent with the hypothesis that the length of the biliopancreatic limb is a relevant determinant of the resulting BA signature.

After BMS, the postprandial FGF19 response was inverted compared with controls, with a progressive increase during the MMT after both techniques. This finding is consistent with the “distal intestine hypothesis”: the anatomical modification allows nutrients and BAs to reach the terminal ileum early, activating the FXR receptor and stimulating FGF19 secretion [6]. FGF19 exerts a dual metabolic action: on the one hand, a potent negative feedback on hepatic BA synthesis through inhibition of CYP7A1 (cholesterol 7-α-hydroxylase), the rate-limiting enzyme of its synthesis; and, on the other, it regulates carbohydrate metabolism (inhibition of gluconeogenesis) and lipid metabolism (suppression of de novo lipogenesis) in addition to acting as a regulator of energy balance. Its strong-to-moderate correlation with the change in conjugated BAs, particularly robust in SADI-S, could be consistent with a more efficient enterohepatic FXR-FGF19 signaling after this technique, which in people with diabetes has been associated with higher remission rates in previous studies [6,24]. Assessment of diabetes remission was beyond the primary aim of the present study, and the number of patients with baseline diabetes did not allow for adequately powered subgroup analyses; this mechanistic association therefore warrants confirmation in future studies specifically designed for this purpose. Correlation does not imply causality, and this association would require confirmation in dedicated mechanistic studies.

Severe obesity was characterized by a significantly lower AUC of total BAs than the control group, which could contribute to reduced FXR/TGR5 signaling and pre-surgical insulin resistance. This baseline deficit is consistent with the dysbiosis described in obesity, with a reduction in bacteria that produce secondary BAs and altered BA transport [12]. After BMS, both techniques increased total BAs, particularly significantly in RYGB. This increase is characteristic of techniques with an intestinal diversion component and is not observed after diet-induced weight loss or after purely restrictive techniques [18,25]. In particular, the unconjugated primary BAs (CA and CDCA), deficient in obesity, recovered after both techniques, reflecting a restoration of the bile acid pool.

Secondary BAs behaved differently depending on the technique. In RYGB, the increase in DCA and UDCA is consistent with studies documenting gradual elevations of DCA at 12–24 months, linked to improved hepatic insulin sensitivity; indeed, UDCA and its conjugates have been identified as potent insulin sensitizers, capable of reducing fasting glucose, HbA1c, and insulin levels [25,26]. In SADI-S, although the increase in the AUC of secondary BAs did not reach the significance seen in RYGB, conjugated forms predominated: fasting TDCA and GUDCA concentrations were higher than in RYGB. These conjugated secondary BAs are associated in the literature with weight reduction through TGR5 activation and with favorable correlations with glucose, HbA1c, and GLP-1, which would be compatible with the hypothesis that the SADI-S anatomy favors greater recirculation and hepatic conjugation of BAs [9,27].

In an exploratory analysis, the increase in conjugated BAs correlated negatively with the percentage of recurrence weight gain at 5 years, which could suggest a role for BAs in long-term weight maintenance, possibly mediated by sustained TGR5 stimulation [7,25]. However, given the number of correlations tested, the moderate sample size, and the absence of multiplicity correction, this finding should be considered hypothesis-generating. Moreover, although weight loss was greater with SADI-S at one year, the differences in %WL at 3 and 5 years and in recurrence weight gain did not reach statistical significance; our data therefore do not demonstrate superior long-term weight outcomes with SADI-S, and the conjugated BA profile should be regarded only as a potential candidate mechanism for weight maintenance. SADI-S, a technique developed at our center [13,27], has shown favorable long-term weight outcomes in previous series; a randomized trial of duodenal switch, the procedure from which SADI-S derives, documented significantly greater weight loss than RYGB (mean difference ≈ 10%) [28], and greater weight loss with SADI-S than with RYGB was also reported in the recent SADISLEEVE randomized trial [29]. Confirmation of a BA-mediated contribution will require adequately powered randomized studies with mechanistic endpoints, as other mechanisms may coexist.

The decrease in the LCA AUC after surgery (significant in RYGB), with values lower than the control group after both techniques, has potential hepatoprotective relevance, given that LCA is the most cytotoxic BA, capable of inducing hepatocyte apoptosis and hepatic inflammation [30,31]. This sustained decrease could reflect a more efficient sequestration or fecal elimination of this molecule, constituting an additional protective mechanism.

Regarding gastrointestinal and pancreatic hormones, the postprandial insulin peak shifted from minute 60 to minute 30 in both techniques, with a markedly different magnitude: 21-fold the baseline value in RYGB versus 14-fold in SADI-S. This difference is possibly related to accelerated gastric emptying, which occurs after both techniques but is more pronounced in the RYGB group due to the absence of a pylorus [32]; in SADI-S, the sleeve gastrectomy also accelerates transit, although less markedly. This early nutrient arrival to the distal intestine would stimulate hormones such as GLP-1 earlier and more intensely. This more abrupt action has been associated with greater susceptibility to postprandial hypoglycemia in RYGB [33,34]. The lower insulin area under the curve in SADI-S may reflect greater metabolic efficiency; that is, the same glycemic control with lower insulin secretion, which may contribute to reduced long-term glycemic variability, as previously demonstrated with continuous glucose monitoring after this technique [27]. The GLP-1 response was supraphysiological in both techniques and, in SADI-S, its increase at 30 min correlated with weight loss at 3 years (an exploratory finding of marginal significance), in line with the literature linking a lower GLP-1 response to poorer weight evolution [35]. The Long-Limb study further demonstrated that lengthening the biliopancreatic limb in RYGB does not additionally increase GLP-1 secretion [36], suggesting that it is the early arrival of nutrients to the distal intestine, and not the limb length itself, that determines the incretin response.

Ghrelin behavior was opposite depending on the technique: fasting concentrations increased after RYGB, in which the gastric fundus is preserved in the excluded stomach, whereas they were suppressed after SADI-S, in which the fundus is resected with the sleeve gastrectomy [5]. The effect of RYGB on ghrelin is controversial, since meta-analyses describe decreased, unchanged, or increased concentrations depending on how the gastric reservoir is sectioned [37], which could explain part of the disparity in the literature. In our cohort, the RYGB gastric pouch was approximately 20–30 mL, although its exact size could vary depending on the surgeon, and the SADI-S sleeve was calibrated over a 54 Fr bougie; inter-individual variability in pouch size and in the extent of fundic resection may therefore have influenced ghrelin dynamics. Other factors, such as changes in gastric emptying, vagal innervation, and the magnitude of weight loss and adiposity, may also modulate ghrelin secretion independently of the presence of fundic tissue, so the difference between techniques cannot be attributed solely to the anatomical difference in the gastric fundus. The suppression of ghrelin secretion observed after SADI-S could represent an advantage for appetite control, although this was not assessed in the present study.

The decrease in leptin was comparable between techniques and showed a strong negative correlation with %WL, consistent with leptin being a marker of total adiposity and with the meta-analysis of adipokines after bariatric surgery [37]; in our exploratory regression analysis, its decrease was associated with weight-loss success at 3 years, a finding that requires confirmation in larger cohorts. The adiponectin/leptin ratio, proposed as an integrated biomarker of adipose dysfunction [10,11], underwent a change of great clinical magnitude, shifting from 90% of patients in the severe-risk range (<0.5) to 99% in the normal range at one year in both surgical techniques, even exceeding the values of the control group. This finding is consistent with that described by Unamuno et al. [38], who documented a significant increase in this ratio after RYGB with normalization of cardiometabolic risk.

Finally, a clinically relevant contribution of this work is that the SeHCAT test ruled out bile acid malabsorption in 100% of SADI-S patients and showed normal retention in 90.6% of RYGB patients. These results indicate that, one year after surgery, neither technique was associated with clinically relevant loss of BAs to the colon, so the changes in the BA profile are unlikely to be explained by bile acid malabsorption [39]. However, SeHCAT specifically assesses the ileal reabsorption of a conjugated BA analog and does not evaluate the absorption of fat, protein, carbohydrates, vitamins, or minerals. Our findings therefore do not exclude a nutritional malabsorptive component of SADI-S, which is consistent with the postoperative decreases in total protein, albumin (in RYGB), and several micronutrients observed in our cohort (Table 2). A possible explanation for the positivity of the SeHCAT test in the few RYGB patients may be related to the frequency of functional exocrine pancreatic insufficiency, reported in these patients at between 10 and 25% [40,41], although not always clinically evident (e.g., steatorrhea).

From a nutritional standpoint, our results have several practical implications. First, the normal SeHCAT retention observed after SADI-S and in most RYGB patients suggests that bile acid loss is unlikely to be the main mechanism of fat-soluble vitamin deficiency after these procedures. Second, despite routine supplementation, total protein, zinc, copper and the vitamin A/RBP and vitamin E/cholesterol ratios decreased after both techniques (Table 2), which supports systematic long-term monitoring of nutritional status and individualized supplementation, regardless of the surgical technique. Finally, the standardized mixed-meal test used in this study provides a physiological nutrient challenge that integrates digestive, enterohepatic and enteroendocrine responses and may be a useful tool to characterize how each technique modifies the handling of nutrients.

Taken together, these results suggest that determination of the BA and gastrointestinal hormone profile may provide hypothesis-generating information: in exploratory analyses, the increase in conjugated BAs was associated with lower recurrence weight gain, and the decrease in leptin with weight-loss success at 3 years. Although their routine determination cannot yet be recommended in clinical practice, these biomarkers could, in the future, help to select the optimal technique or to promptly identify those patients at risk of recurrence weight gain. If confirmed, these findings could contribute to a better understanding of the role of enterohepatic BA signaling together with gastrointestinal and adipose-tissue hormones, in the metabolic effects of malabsorptive techniques.

This study has some limitations, as it is a single-center study, which limits comparison with other populations. The non-randomized observational design entails baseline differences between groups that could introduce bias in the direct comparisons. In particular, SADI-S patients had a higher baseline BMI and RYGB patients a numerically higher prevalence of T2DM, both of which may influence BA and hormonal patterns. Given the moderate sample size and the number of outcomes examined, no multivariable adjustment for baseline BMI, T2DM status, age, sex or baseline BA levels was performed; between-group differences should therefore be interpreted as associations rather than as causal effects of specific anatomical features. Many comparisons were performed across BA fractions, hormones, time points, and groups; although FDR correction was applied to the individual BA analyses, the remaining analyses, including correlations and logistic regression, are based on nominal *p* values and should be considered exploratory, with a risk of false-positive findings. The logistic regression analysis was not based on a prespecified variable-selection strategy and formal model diagnostics were not performed; given the sample size, there is a risk of overfitting, and the association of leptin with weight-loss success requires confirmation. Long-term weight analyses were performed on a complete-case basis without imputation; although retention was high (85% at 5 years), a formal comparison between participants retained and lost to follow-up was not performed, and attrition related to weight trajectory cannot be excluded. Several analyses originally planned within the FIS-funded project—namely gut microbiota profiling, intraoperative liver biopsy biomarkers, and PYY quantification—are not reported here; all of them were collected as part of the original protocol and will be presented in dedicated future publications addressing, respectively, the microbiota–bile acid axis, MASLD-related hepatic biomarkers, and the appetite-regulating hormonal axis after each surgical technique. As a consequence, a fully integrated view of the gut–liver–microbiota axis, a key pathophysiological mechanism, is beyond the scope of the present work. The absence of PYY and gut microbiota data in the present manuscript limits the characterization of the gut hormone response and of the microbiota–bile acid axis, which is a key determinant of secondary BA formation and deconjugation. Quantitative adherence to postoperative micronutrient supplementation was not systematically recorded, and non-uniform adherence may have contributed to the variability of the nutritional and micronutrient results (Table 2), although it is less likely to affect the BA and hormonal findings. Inter-individual variability in gastric pouch size and in the extent of fundic resection may have influenced ghrelin dynamics. Finally, ^75^Se-SeHCAT assesses only bile acid retention and does not allow conclusions regarding the absorption of other nutrients. Determinations were performed only one year after BMS, so short- or longer-term results are not fully explored. An additional consideration is that the course of the study coincided with the COVID-19 pandemic, which caused a substantial delay in sample collection and processing. However, this study also has notable strengths: the prospective design with paired pre- and post-surgical assessment in each patient, the inclusion of a healthy non-obese control group, the quantification of 14 individual BA fractions by UHPLC-MS rather than total BAs alone, the use of a dynamic mixed-meal test instead of fasting measurements only, the objective assessment of bile acid malabsorption by ^75^Se-SeHCAT, and a weight follow-up extending to five years.

## 5. Conclusions

In conclusion, SADI-S and RYGB are associated with distinct BA and hormonal profiles one year after surgery. SADI-S was associated with higher fasting concentrations of conjugated BAs, which we hypothesize may be related to the greater length of its biliopancreatic limb, whereas RYGB showed a greater postprandial increase in the unconjugated secondary BA DCA. The two techniques also showed differentiated hormonal responses: an earlier postprandial insulin peak after both procedures, more pronounced after RYGB, with a lower insulin AUC after SADI-S; supraphysiological GLP-1 responses; and opposite changes in ghrelin, which increased after RYGB and was suppressed after SADI-S. These hormonal changes probably act together with BA changes in the metabolic improvement after surgery. In exploratory analyses, the increase in conjugated BAs was negatively associated with recurrence weight gain at 5 years, pointing to enterohepatic BA signaling as a potential candidate mechanism for weight maintenance, a hypothesis that requires confirmation in adequately powered randomized studies. The ^75^Se-SeHCAT test showed no clinically relevant bile acid malabsorption after either technique, although this test does not assess the absorption of other nutrients. From a nutritional perspective, the absence of bile acid malabsorption, together with the decreases in several micronutrients observed after both techniques despite routine supplementation, supports systematic nutritional monitoring and individualized supplementation after malabsorptive metabolic surgery.

## Figures and Tables

**Table 1:** The 14 individual bile acids quantified in the study, grouped by origin (primary/secondary) and conjugation status.

Bile Acid	Abbreviation	Notes
Primary bile acids		
Cholic acid	CA	Unconjugated
Chenodeoxycholic acid	CDCA	Unconjugated
Glycocholic acid	GCA	Conjugated with glycine
Glycochenodeoxycholic acid	GCDCA	Conjugated with glycine
Taurocholic acid	TCA	Conjugated with taurine
Taurochenodeoxycholic acid	TCDCA	Conjugated with taurine
Secondary bile acids		
Deoxycholic acid	DCA	Unconjugated
Lithocholic acid	LCA	Unconjugated
Ursodeoxycholic acid	UDCA	Unconjugated
Glycodeoxycholic acid	GDCA	Conjugated with glycine
Glycoursodeoxycholic acid	GUDCA	Conjugated with glycine
Taurodeoxycholic acid	TDCA	Conjugated with taurine
Tauroursodeoxycholic acid	TUDCA	Conjugated with taurine
Taurolithocholic acid	TLCA	Conjugated with taurine

Primary bile acids are synthesized in the liver from cholesterol; secondary bile acids are generated from primary bile acids through microbial 7α-dehydroxylation and epimerization in the intestine. Both primary and secondary bile acids may circulate in unconjugated form or conjugated with glycine or taurine.

**Table 2:** Evolution of biochemical parameters (glycemic, lipid, hepatic, nutritional, and micronutrient profiles) before and one year after bariatric–metabolic surgery, according to surgical technique.

	RYGB			SADI-S		
Parameter	Pre	1 Year	*p*	Pre	1 Year	*p*
Glycemic profile (mean ± SD)						
Glucose (mg/dL)	106.0 ± 38.2	86.1 ± 15.4	<0.01	122.4 ± 46.8	91.3 ± 26.0	<0.01
HbA1c (%)	6.1 ± 1.3	5.3 ± 0.5	<0.01	6.5 ± 1.7	5.3 ± 0.8	<0.01
HOMA-IR	9.1 ± 6.2	2.13 ± 1.9	<0.01	8.9 ± 6.0	1.62 ± 1.1	<0.01
Lipid profile (mean ± SD)						
Total cholesterol (mg/dL)	186.6 ± 30.0	154.0 ± 33.8	<0.05	191.8 ± 38.2	153.6 ± 28.4	<0.05
HDL-C (mg/dL)	48.7 ± 13.0	57.1 ± 11.5	<0.05	48.4 ± 12.2	53.5 ± 12.1	<0.05
LDL-C (mg/dL)	111.9 ± 27.9	80.9 ± 26.0	<0.05	117.9 ± 33.0	85.7 ± 23.8	<0.05
Triglycerides (mg/dL)	123.9 ± 48.2	80.1 ± 23.8	<0.05	141.0 ± 57.4	77.4 ± 24.6	<0.05
Hepatic profile (mean ± SD)						
AST (U/L)	22.3 ± 7.9	24.6 ± 7.7	n.s.	23.0 ± 8.4	26.5 ± 8.5	n.s.
ALT (U/L)	25.6 ± 16.6	26.1 ± 11.7	n.s.	27.1 ± 16.0	28.6 ± 15.3	n.s.
ALP (U/L)	83.9 ± 21.1	97.2 ± 22.2	n.s.	84.2 ± 23.6	99.0 ± 30.0	n.s.
GGT (U/L)	33.3 ± 18.2	17.2 ± 8.5	<0.05	33.0 ± 14.2	28.8 ± 12.2	<0.05
Nutritional and micronutrient profile (median [IQR])						
Hemoglobin (g/dL)	12.8 (9.8–16.0)	12.2 (7.9–14.0)	0.531	13.1 (8.9–14.5)	12.0 (7.7–14.8)	0.611
Total protein (g/dL)	6.95 (6.62–7.10)	6.40 (6.20–6.57)	<0.001	7.10 (6.90–7.30)	6.60 (6.20–7.00)	<0.001
Albumin (g/dL)	4.10 (3.92–4.20)	3.90 (3.80–4.10)	0.026	4.00 (3.90–4.20)	3.90 (3.90–4.10)	0.213
Iron (µg/dL)	71.0 (61.0–95.5)	75.5 (50.5–97.5)	0.778	63.0 (50.5–79.5)	88.0 (61.5–107.5)	<0.001
Ferritin (ng/mL)	59.4 (19.9–106.1)	59.1 (19.8–100.6)	0.522	64.4 (30.9–83.0)	70.8 (32.0–116.4)	0.580
Folate (ng/mL)	6.43 (4.66–8.97)	9.68 (6.38–13.19)	0.011	4.88 (3.92–6.87)	4.18 (2.60–5.93)	0.055
Vitamin B12 (pg/mL)	256 (196–299)	222 (168–302)	0.170	262 (230–306)	301 (228–370)	0.320
PTH (pg/mL)	68.6 (53.3–84.6)	60.7 (53.0–73.6)	0.089	62.4 (52.0–84.9)	75.6 (63.8–85.4)	0.096
25-OH Vitamin D (ng/mL)	14.7 (10.9–18.8)	19.8 (16.7–27.4)	<0.001	16.0 (12.3–31.0)	17.4 (11.4–28.8)	0.924
Vitamin A/RBP (mg/mg)	0.796 (0.730–0.880)	0.725 (0.670–0.800)	<0.05	0.793 (0.710–0.920)	0.710 (0.670–0.790)	<0.05
Vitamin E/total cholesterol (mg/g)	7.95 (6.52–8.77)	6.10 (5.50–6.90)	<0.01	7.45 (6.10–8.90)	5.90 (5.40–7.40)	<0.001
Zinc (µg/dL)	87.5 (78.7–95.5)	76.2 (68.2–86.1)	<0.01	86.2 (81.1–94.4)	75.2 (65.5–82.5)	<0.01
Copper (µg/dL)	128.0 (121.5–148.5)	118.0 (106.7–132.2)	<0.01	126.2 (114.5–132.6)	102.0 (85.6–125.3)	<0.01

Values are expressed as mean ± standard deviation (SD) for the glycemic, lipid, and hepatic profiles (normally distributed variables), and as median (interquartile range, IQR) for the nutritional and micronutrient profile (non-normally distributed variables). *p* values correspond to within-group comparison before vs. 1 year after surgery, using the paired Student’s *t*-test or the Wilcoxon signed-rank test, as appropriate. Statistically significant values (*p* < 0.05) are shown in bold. n.s., not significant. Abbreviations: RYGB, Roux-en-Y gastric bypass; SADI-S, single-anastomosis duodeno-ileal bypass with sleeve gastrectomy; HbA1c, glycated hemoglobin; HOMA-IR, homeostatic model assessment of insulin resistance; HDL-C, high-density lipoprotein cholesterol; LDL-C, low-density lipoprotein cholesterol; AST, aspartate aminotransferase; ALT, alanine aminotransferase; ALP, alkaline phosphatase; GGT, gamma-glutamyl transferase; PTH, parathyroid hormone; RBP, retinol-binding protein.

**Table 3:** Baseline demographic characteristics of the study population.

Parameter	Controls	Before Surgery	*p* ^1^	RYGB	SADI-S	*p* ^2^
Total, n (%)	19	68		37	31	
Age (years)	48.4 ± 9.53	47.9 ± 9.74	n.s.	50.0 ± 9.25	45.4 ± 9.85	0.051
Sex (women), n (%)	18 (95)	58 (85.3%)	<0.01	31 (83.8%)	27 (84.4%)	1.000
Preoperative weight (kg)	57.9 ± 8.5	120 ± 19.8	<0.01	115 ± 19.7	127 ± 18.3	0.015
BMI (kg/m^2^)	23 ± 2.43	45.9 ± 4.86	<0.01	44.1 ± 4.28	48.0 ± 4.72	<0.001
Waist circumference (cm)	80.8 ± 9.9	133 ± 12.3	<0.01	131 ± 10.1	135 ± 14.4	0.160
Hypertension, n (%)	0	26 (38.2%)	<0.01	16 (43.2%)	10 (31.2%)	0.438
Type 2 diabetes mellitus, n (%)	0	35 (51.5%)	<0.01	22 (59.5%)	13 (40.6%)	0.187
Hyperlipidemia, n (%)	0	22 (32.4%)	<0.01	14 (37.8%)	8 (25.0%)	0.378
OSA, n (%)	0	13 (19.1%)	<0.01	7 (18.9%)	6 (18.8%)	1.000

Data expressed as mean ± (SD) or percentages. *p* ^1^: differences between the control group and all surgical patients; *p* ^2^: differences between surgical techniques (RYGB vs. SADI-S). N: number; SD: standard deviation; BMI: body mass index; OSA: obstructive sleep apnea. n.s., not significant.

**Table 4:** Weight follow-up before RYGB and SADI-S, and after at 1, 3, and 5 years.

Parameter	RYGB	SADI-S	*p*	Follow-Up
BMI pre (kg/m^2^)	44.1 ± 4.28	48.0 ± 4.72	<0.001	—
BMI 1 year (kg/m^2^)	28.4 ± 3.66	28.0 ± 6.01	n.s.	100%
BMI 3 years (kg/m^2^)	27.6 ± 3.85	29.6 ± 3.60	n.s.	97%
BMI 5 years (kg/m^2^)	29.1 ± 3.40	30.7 ± 4.20	n.s.	85%
%WL 1 year	35.4 ± 7.77	41.3 ± 12.8	0.028	100%
%WL 3 years	37.1 ± 7.90	39.0 ± 6.50	n.s.	97%
%WL 5 years	33.8 ± 7.30	37.1 ± 8.30	n.s.	85%
Weight regain from nadir at 5 years (%)	14.9 (95% CI: 3.6–18.1)	10.4 (95% CI: −0.6–19.1)	n.s.	—

%WL: percentage of weight loss; BMI: body mass index. *p*: comparison between RYGB and SADI-S; n.s.: not significant. Follow-up: follow-up rate at each time point.

**Table 5:** Fasting concentrations and area under the curve (AUC) of gastrointestinal, pancreatic, and adipose-tissue hormones before and one year after bariatric–metabolic surgery, compared with the control group.

Hormone	Controls	RYGB		SADI-S		*p* ^1^ RYGB	*p* ^1^ SADI-S	*p* ^2^
		Pre	1 Year	Pre	1 Year			
Fasting insulin (µIU/mL)	6.6 ± 2.85	35.8 ± 29.6	7.6 ± 7.0	41.3 ± 50.1	5.81 ± 3.45	<0.01	<0.01	n.s.
Insulin AUC (µIU·mL^−1^·min)	6104 ± 2417	11,729 ± 5655	8962 ± 4416	12,546 ± 6582	5092 ± 2843	<0.01	<0.01	<0.01
Fasting GLP-1 (pmol/L)	20.2 ± 8.3	41.5 ± 34.7	30.6 ± 33.5	47.5 ± 20.3	26.8 ± 14.1	<0.05	<0.01	n.s.
GLP-1 AUC (pmol·L^−1^·min)	2,778 ± 570	5715 ± 4559	12,542 ± 4284	6266 ± 2222	10,540 ± 3029	<0.01	<0.01	<0.05
Fasting ghrelin (pmol/L)	481.8 ± 282.5	259.4 ± 141.2	349.0 ± 205.6	205.3 ± 233.6	139.7 ± 100.1	<0.05	n.s.	<0.01
Ghrelin AUC (pmol·L^−1^·min)	40,289 ± 18,117	26,068 ± 9742	27,875 ± 15,522	25,531 ± 21,928	14,261 ± 7848	n.s.	<0.01	<0.01
Fasting FGF19 (pg/mL)	130.4 ± 86.2	144.6 ± 342.9	179.6 ± 217.1	115.4 ± 94.5	223.1 ± 148.4	n.s.	<0.01	n.s.
FGF19 AUC (pg·mL^−1^·min)	17,528 ± 9772	16,835 ± 32,531	29,970 ± 38,400	14,006 ± 7537	34,723 ± 22,564	<0.05	<0.01	n.s.
Leptin (ng/mL)	9.3 ± 6.5	34.9 ± 34.8	5.4 ± 5.7	30.6 ± 15.8	6.6 ± 5.8	<0.01	<0.01	n.s.
Adiponectin (µg/mL)	10.0 ± 4.1	6.4 ± 5.0	11.7 ± 10.6	6.3 ± 4.3	11.0 ± 7.6	<0.01	<0.01	n.s.

Values expressed as mean ± standard deviation (SD). *p* ^1^: comparison before vs. one year after surgery, within each technique (paired Student’s *t*-test or Wilcoxon signed-rank test as appropriate). *p* ^2^: comparison between RYGB and SADI-S at one year (Mann–Whitney U or independent *t*-test as appropriate). Significant values (*p* < 0.05) are shown in bold. n.s., not significant. Abbreviations: RYGB, Roux-en-Y gastric bypass; SADI-S, single-anastomosis duodeno-ileal bypass with sleeve gastrectomy; AUC, area under the curve; GLP-1, glucagon-like peptide 1; FGF19, fibroblast growth factor 19.

**Table 6:** Total, primary, and secondary bile acids (AUC).

Bile Acid	Controls	Pre-Surgery RYGB	Pre-Surgery SADI-S	RYGB 1 Year	*p* ^1^ RYGB	SADI-S 1 Year	*p* ^1^ SADI-S	*p* ^2^
Total BAs—AUC (mg·L^−1^·min)	383.1 (294.8–476.8)	211.2 (149.6–410.8)	269.3 (193.8–426.9)	324.4 (225.1–572.1)	<0.05	349.8 (222.8–566.4)	n.s.	n.s.
Primary BAs—AUC (mg·L^−1^·min)	228.9 (176.0–284.8)	148.7 (79.1–266.5)	194.9 (109.9–290.5)	202.4 (139.8–353.3)	n.s.	222.9 (141.7–371.1)	n.s.	n.s.
Secondary BAs—AUC (mg·L^−1^·min)	140.5 (85.3–192.4)	86.2 (48.1–124.5)	79.1 (70.0–135.4)	137.6 (91.9–211.0)	<0.05	108.8 (64.4–211.2)	n.s.	n.s.

Data expressed as median (interquartile range). *p* ^1^: comparison before vs. one year after surgery within each technique (Wilcoxon signed-rank test); *p* ^2^: comparison between RYGB and SADI-S at one year (Mann–Whitney U test). n.s., not significant.

**Table 7:** Conjugated primary and secondary bile acids (fasting and AUC).

Bile Acid	Controls	Pre-Surgery RYGB	Pre-Surgery SADI-S	RYGB 1 Year	*p* ^1^ (q) RYGB	SADI-S 1 Year	*p* ^1^ (q) SADI-S	*p* ^2^ (q)
***CONJUGATED PRIMARY***								
GCA—fasting (mg/L)	0.05 (0.03–0.06)	0.04 (0.02–0.06)	0.05 (0.03–0.11)	0.15 (0.08–0.40)	**<0.001** **(<0.001)**	0.34 (0.14–0.89)	**<0.001** **(<0.001)**	**0.010** **(0.046)**
GCA—AUC (mg·L^−1^·min)	33.3 (10.9–39.0)	13.0 (7.5–18.7)	18.2 (9.3–31.5)	18.0 (9.4–30.6)	0.190 (0.332)	42.9 (14.0–77.8)	0.004 (0.055)	0.026 (0.119)
GCDCA—fasting (mg/L)	0.17 (0.08–0.28)	0.15 (0.08–0.24)	0.25 (0.17–0.40)	0.35 (0.21–0.85)	**<0.001** **(<0.001)**	1.2 (0.53–2.68)	**<0.001** **(<0.001)**	**<0.001** **(0.014)**
GCDCA—AUC (mg·L^−1^·min)	112.1 (71.8–150.6)	83.1 (40.5–111.1)	79.0 (63.8–129.9)	84.4 (36.8–140.2)	0.822 (0.861)	124.1 (58.1–169.8)	0.148 (0.255)	0.076 (0.212)
TCDCA—fasting (mg/L)	0.012 (0.009–0.020)	0.016 (0.006–0.036)	0.022 (0.013–0.065)	0.022 (0.009–0.050)	0.230 (0.248)	0.045 (0.014–0.138)	0.032 (0.074)	0.043 (0.100)
TCDCA—AUC (mg·L^−1^·min)	11.1 (4.4–17.4)	7.7 (1.3–14.2)	9.9 (3.9–11.6)	3.0 (1.4–11.2)	0.408 (0.520)	6.8 (3.9–11.6)	0.413 (0.526)	0.037 (0.129)
TCA—fasting (mg/L)	0.006 (0.002–0.020)	0.009 (0.004–0.03)	0.023 (0.006–0.035)	0.014 (0.005–0.028)	0.123 (0.156)	0.011 (0.005–0.025)	0.234 (0.364)	0.685 (0.738)
TCA—AUC (mg·L^−1^·min)	2.6 (1.1–3.8)	3.0 (1.2–4.7)	3.7 (2.1–8.5)	1.7 (1.1–3.8) ↓	0.499 (0.582)	1.5 (0.8–3.9) ↓	0.016 (0.113)	0.556 (0.787)
***CONJUGATED SECONDARY***								
GDCA—fasting (mg/L)	0.04 (0.03–0.12)	0.05 (0.02–0.08)	0.06 (0.04–0.15)	0.22 (0.11–0.34)	**<0.001** **(<0.001)**	0.41 (0.13–0.69)	**<0.001** **(<0.001)**	0.094 (0.164)
GDCA—AUC (mg·L^−1^·min)	28.7 (24.9–75.5)	25.9 (7.4–41.2)	22.2 (15.6–38.9)	27.0 (16.6–69.3)	0.080 (0.159)	27.2 (15.4–86.3)	0.239 (0.334)	0.755 (0.881)
TDCA—fasting (mg/L)	0.01 (0.004–0.014)	0.006 (0.004–0.016)	0.017 (0.006–0.031)	0.014 (0.008–0.024)	0.063 (0.088)	0.032 (0.010–0.070)	**0.010** **(0.036)**	**0.006** **(0.043)**
TDCA—AUC (mg·L^−1^·min)	8.1 (2.2–10.5)	3.0 (1.8–6.7)	3.9 (2.0–8.0)	1.7 (0.9–4.5)	0.280 (0.435)	2.5 (1.4–5.7)	0.164 (0.255)	0.294 (0.570)
GUDCA—fasting (mg/L)	0.03 (0.02–0.05)	0.013 (0.007–0.03)	0.03 (0.02–0.07)	0.044 (0.02–0.06)	**0.016** **(0.038)**	0.08 (0.03–0.20)	**0.014** **(0.040)**	0.040 (0.100)
GUDCA—AUC (mg·L^−1^·min)	9.5 (7.0–11.9)	4.4 (2.1–12.3)	6.1 (3.6–11.9)	3.0 (1.9–7.4)	0.394 (0.520)	5.6 (3.8–9.9)	0.934 (0.934)	0.014 (0.098)
TUDCA—fasting (mg/L)	0.005 (0.004–0.005)	0.005 (0.003–0.01)	0.005 (0.004–0.009)	0.008 (0.005–0.010)	0.161 (0.188)	0.005 (0.003–0.009)	0.897 (0.897)	0.120 (0.187)
TUDCA—AUC (mg·L^−1^·min)	2.3 (0.8–6.0)	0.85 (0.45–1.5)	0.83 (0.48–1.88)	0.92 (0.5–2.3)	0.861 (0.861)	0.92 (0.5–2.0)	0.878 (0.934)	1.000 (1.000)
TLCA—fasting (mg/L)	0.007 (0.006–0.010)	0.002 (0.001–0.03)	0.003 (0.001–0.03)	0.011 (0.004–0.030)	**0.003** **(0.010)**	0.007 (0.004–0.013)	0.509 (0.593)	0.484 (0.616)
TLCA—AUC (mg·L^−1^·min)	3.4 (2.5–8.0)	0.32 (0.14–1.0)	0.5 (0.23–1.93)	1.4 (0.6–4.0) ↑	**0.005** **(0.036)**	1.0 (0.5–2.0) ↑	0.150 (0.255)	0.909 (0.979)

Data expressed as median (interquartile range). *p* ^1^: comparison before vs. one year after surgery within each technique (Wilcoxon signed-rank test; paired data available for *n* = 26 RYGB and *n* = 26–28 SADI-S patients). *p* ^2^: comparison between RYGB and SADI-S at one year (Mann–Whitney U test). Values in parentheses are Benjamini–Hochberg false discovery rate (FDR)-adjusted q values, calculated within each family of 14 bile acid fractions, separately for fasting concentrations and AUCs. Values in bold: q < 0.05. Arrows indicate the direction of change.

**Table 8:** Unconjugated primary and secondary bile acids (fasting and AUC).

Bile Acid	Controls	Pre-Surgery RYGB	Pre-Surgery SADI-S	RYGB 1 Year	*p* ^1^ (q) RYGB	SADI-S 1 Year	*p* ^1^ (q) SADI-S	*p* ^2^ (q)
***UNCONJUGATED PRIMARY***								
CA—fasting (mg/L)	0.12 (0.08–0.30)	0.035 (0.02–0.14)	0.04 (0.02–0.20)	0.21 (0.05–0.78)	**0.004** **(0.010)**	0.10 (0.04–0.40)	0.100 (0.185)	0.386 (0.540)
CA—AUC (mg·L^−1^·min)	10.7 (5.8–47.0)	5.5 (2.0–14.1)	3.9 (1.5–13.8)	16.9 (6.6–41.2)	**0.009** **(0.040)**	13.6 (5.3–29.2)	0.129 (0.255)	0.562 (0.787)
CDCA—fasting (mg/L)	0.37 (0.23–0.58)	0.14 (0.067–0.42)	0.17 (0.07–0.46)	0.48 (0.20–1.50)	**0.027** **(0.042)**	0.32 (0.13–0.96)	0.106 (0.185)	0.544 (0.635)
CDCA—AUC (mg·L^−1^·min)	57.4 (19.3–74.0)	17.4 (9.4–73.3)	25.6 (11.0–68.3)	63.1 (27.5–98.9)	0.043 (0.108)	40.0 (19.4–82.0)	0.155 (0.255)	0.300 (0.570)
***UNCONJUGATED SECONDARY***								
DCA—fasting (mg/L)	0.25 (0.12–0.36)	0.17 (0.10–0.34)	0.20 (0.12–0.40)	0.38 (0.20–0.90)	**0.020** **(0.041)**	0.22 (0.14–0.30)	0.485 (0.593)	0.039 (0.100)
DCA—AUC (mg·L^−1^·min)	39.5 (26.0–58.9)	27.4 (14.6–40.8)	19.9 (13.7–45.1)	61.9 (33.8–81.6)	**<0.001** **(<0.001)**	28.9 (14.5–47.4)	0.804 (0.934)	0.004 (0.054)
LCA—fasting (mg/L)	0.012 (0.010–0.040)	0.02 (0.01–0.03)	0.02 (0.015–0.03)	0.020 (0.010–0.040)	0.585 (0.585)	0.020 (0.010–0.040)	0.347 (0.486)	0.742 (0.742)
LCA—AUC (mg·L^−1^·min)	6.2 (1.8–15.6)	2.4 (1.5–3.9)	2.6 (1.8–3.5)	1.6 (0.6–2.5) ↓	0.027 (0.095)	1.8 (0.8–3.5) ↓	0.116 (0.255)	0.325 (0.570)
UDCA—fasting (mg/L)	0.06 (0.04–0.06)	0.03 (0.02–0.08)	0.04 (0.02–0.08)	0.07 (0.04–0.30)	**0.025** **(0.042)**	0.045 (0.03–0.08)	0.728 (0.784)	0.050 (0.100)
UDCA—AUC (mg·L^−1^·min)	10.9 (6.0–26.7)	3.6 (1.9–14.5)	5.0 (2.6–10.8)	11.1 (6.2–31.0)	0.046 (0.108)	9.3 (5.6–16.5)	0.049 (0.229)	0.674 (0.858)

Data expressed as median (interquartile range). *p* ^1^: comparison before vs. one year after surgery within each technique (Wilcoxon signed-rank test; paired data available for *n* = 26 RYGB and *n* = 26–28 SADI-S patients). *p* ^2^: comparison between RYGB and SADI-S at one year (Mann–Whitney U test). Values in parentheses are Benjamini–Hochberg false discovery rate (FDR)-adjusted q values, calculated within each family of 14 bile acid fractions, separately for fasting concentrations and AUCs. Values in bold: q < 0.05. Arrows indicate the direction of change.

**Table 9:** Results of the ^75^Se-SeHCAT isotopic test one year after surgery.

Result	RYGB (*n* = 32)	SADI-S (*n* = 29)
Normal (>15% retention)	29 (90.6%)	29 (100%)
Mild malabsorption (>10% to 15%)	2 (6.25%)	0
Moderate malabsorption (5% to 10%)	1 (3.1%)	0
Severe malabsorption (<5% retention)	0	0

Bile acid malabsorption categories according to ^75^Se-SeHCAT retention at day 7: normal >15%; mild >10% to 15%; moderate 5% to 10%; severe <5% [16,17].

## Data Availability

The raw data supporting the conclusions of this article will be made available by the authors, without undue reservation.
